# Transcriptional Changes following Long-Term Sensitization Training and *In Vivo* Serotonin Exposure in *Aplysia californica*

**DOI:** 10.1371/journal.pone.0047378

**Published:** 2012-10-09

**Authors:** Kristine Bonnick, Karla Bayas, Dmitry Belchenko, Ashly Cyriac, Michael Dove, Jamie Lass, Benora McBride, Irina E. Calin-Jageman, Robert J. Calin-Jageman

**Affiliations:** Neuroscience Program, Dominican University, River Forest, Illinois, United States of America; University of Toronto, Canada

## Abstract

We used *Aplysia californica* to compare the transcriptional changes evoked by long-term sensitization training and by a treatment meant to mimic this training, *in vivo* exposure to serotonin. We focused on 5 candidate plasticity genes which are rapidly up-regulated in the *Aplysia* genus by *in vivo* serotonin treatment, but which have not yet been tested for regulation during sensitization: CREB1, matrilin, antistasin, eIF3e, and BAT1 homolog. CREB1 was rapidly up-regulated by both treatments, but the regulation following training was transient, falling back to control levels 24 hours after training. This suggests some caution in interpreting the proposed role of CREB1 in consolidating long-term sensitization memory. Both matrilin and eIF3e were up-regulated by *in vivo* serotonin but not by long-term sensitization training. This suggests that *in vivo* serotonin may produce generalized transcriptional effects that are not specific to long-term sensitization learning. Finally, neither treatment produced regulation of antistasin or BAT1 homolog, transcripts regulated by *in vivo* serotonin in the closely related *Aplysia kurodai*. This suggests either that these transcripts are not regulated by experience, or that transcriptional mechanisms of memory may vary within the *Aplysia* genus.

## Introduction

Learning produces long-term changes in behavior at least in part through long-lasting changes in neural transcription. Although this principle is now well-documented across the animal kingdom [1]–[3], the transcriptional events that mediate long-term memory are currently a subject of intense inquiry. Specifically, work in a number of model systems has focused on identifying transcripts that are regulated during or immediately after encoding, as these *candidate plasticity genes* could serve to coordinate the subsequent cellular and network changes that reconfigure behavior [4], [5].

Several candidate plasticity genes have been identified through work in *Aplysia*, a genus of mollusks which has proven useful for linking neural and behavioral phenomena. One particular focus has been long-term sensitization, a learning paradigm in which repeated exposure to a noxious stimulus produces a long-lasting and transcription-dependent increase in reflex responsiveness [6], [7]. Behavioral sensitization is thought to require the release of serotonin (5-hydroxytryptamine, 5-HT) during induction [8]. Conveniently, simply exposing intact animals to 5-HT (*in vivo* 5-HT exposure [9]) can mimic many aspects of long-term sensitization training, producing similar transcriptional, cellular, and behavioral effects [10]–[12].

Several candidate plasticity genes have been identified in *Aplysia* which are transcriptionally regulated immediately after *in vivo* 5-HT exposure (see Table 1). In the species *Aplysia californica* these include homologs of C/EBP [9] and CREB1 [13], both of which have been confirmed to play an essential role in the long-term facilitation that accompanies 5-HT exposure [9], [13]. More recent work [14] in the closely related species *Aplysia kurodai* used an EST microarray and qPCR to identify and confirm 4 novel candidate plasticity genes, showing that *in vivo* 5-HT produces an up-regulation in matrilin, antistasin, and eIF3e, and a down-regulation in BAT1 homolog (these names represent ESTs identified in *A. kurodai*; they were named for the GenBank entry with the best BLASTX match to each EST; we maintain that naming convention here; see Table S1). Of these transcripts, eIF3e was further examined and found to serve an essential role in the synaptic changes that accompany 5-HT exposure [14].

**Table 1:** Summary of previous research on regulation of selected Aplysia transcripts by in vivo 5-HT and long-term sensitization training.

Transcript	Regulation by *in vivo* 5-HT^1^	Regulation by long-term sensitization training
**Transcripts of Interest**		
antistasin	Up-regulated immediately in *A. kurodai* [14]	Unknown
matrilin	Up-regulated immediately in *A. kurodai* [14]	Unknown
BAT1 homolog	Down-regulated immediately in *A. kurodai* [14]	Unknown
eIF3e	Up-regulated immediately in *A. kurodai* [14]	Unknown
CREB1	Up-regulated immediately and for at least 12 hours^2^ in *A. californica* [13]	Unknown
**Positive and Negative Controls**		
C/EBP	Up-regulated immediately in *A. californica* [9] and *A. kurodai* [14]	Increased protein expression 1 hour after training [11]
BiP/GRP78	Unknown	Delayed but persistent up-regulation [22]
α-Tubulin 2	Not regulated by *in vivo* 5-HT in *A. kurodai* [14]	Unknown

1: For CREB1, *in vivo* 5-HT exposure was 1 hour at 50 µM; for all other transcripts it was 2 hours at 250 µM.

2: Also shows immediate and long-lasting up-regulation following pulsed 5-HT exposure in isolated ganglia [21], though one report indicates a delayed onset of regulation [30].

Surprisingly, the behavioral relevance of these candidate plasticity genes is not well established. To date, only C/EBP has been shown to be regulated by long-term sensitization training [11]. This is unfortunate because 5-HT treatment paradigms may have significant limitations as models of natural events that produce behavioral sensitization. For example, a recent comparison of long-term sensitization training with 5-HT exposure in isolated ganglia showed that these manipulations produce somewhat different patterns of change in protein expression [15].

In this study, we examined transcriptional regulation of several candidate plasticity genes (CREB1, matrilin, antistasin, eIF3e, and BAT1 homolog) after both long-term sensitization training and *in vivo* 5-HT exposure. This allowed evaluation of a) the behavioral regulation of these transcripts and b) the validity of *in vivo* 5-HT as a model of the transcriptional effects of long-term sensitization training.

## Results

### Transcriptional changes after long-term sensitization training

**Figure 1:**
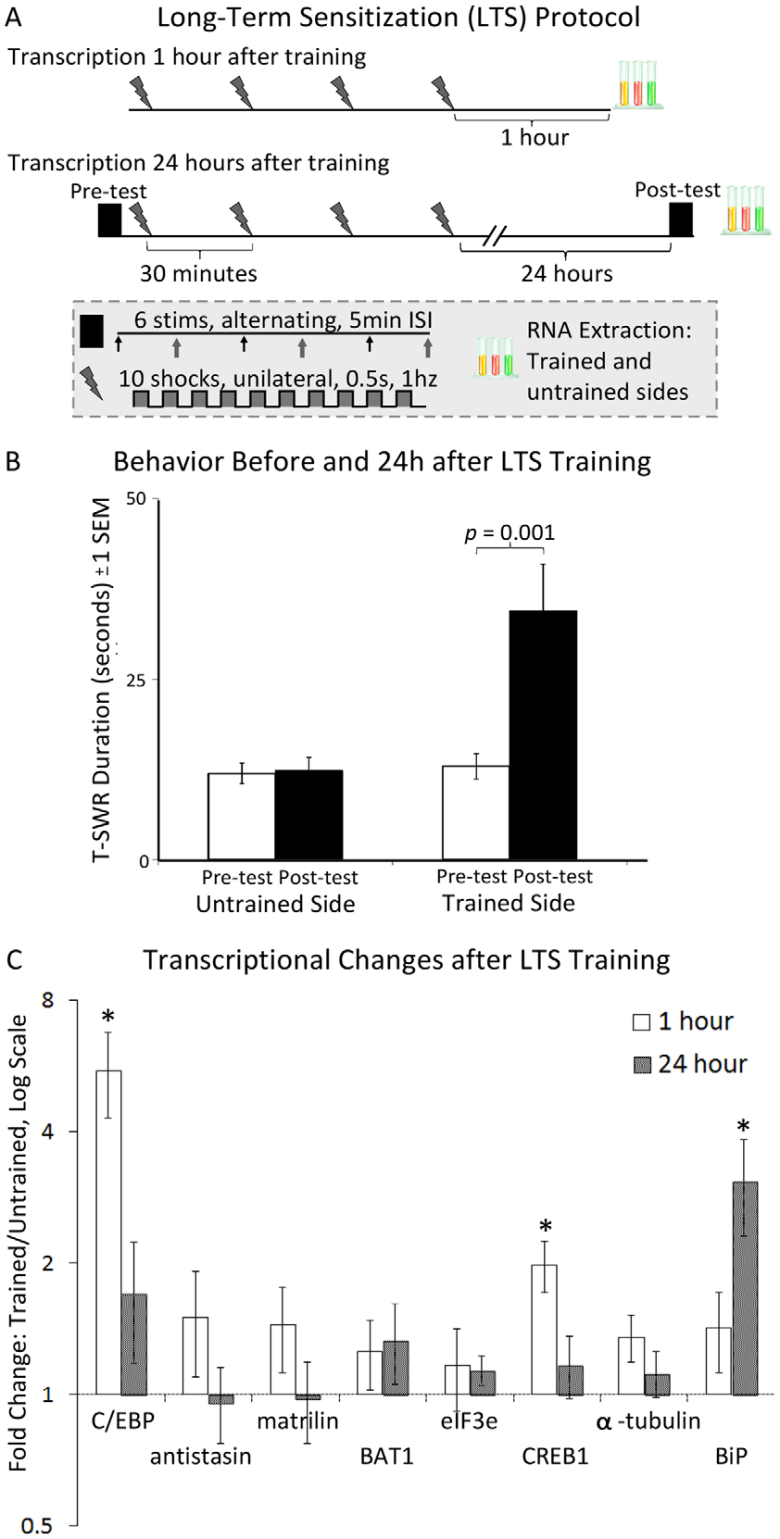
Transcriptional changes following long-term sensitization training. A. Long-term sensitization training protocol. Training consisted of 4 rounds of shock (30 minute interval). In each round, a 10 s shock (90mA AC, 0.5 s on, 0.5 s off) was applied to one side of the body. Pleural ganglia from the trained and untrained side were harvested separately 1 or 24 hours after training ended for qPCR analysis. In the 24-hour group, T-SWR duration was characterized before (pre-test) and 24 hours after (post-test) training. B. Mean T-SWR durations (±1 *SEM*) before and 24 hours after long-term sensitization in the 24 hour group (n = 14). T-SWRs were evoked via weak electrical shock to implanted electrodes in the tail and measured from the time of siphon contraction to the first sign of siphon relaxation. For each animal, pre-test and post-test responding was measured on the trained and untrained side separately as the mean of 3 T-SWRs. The *p* value shown is for a paired t-test comparing pre-test and post-test responses on the trained side. The comparison on the untrained side was not significant. C. Mean transcriptional changes (± 1*SEM*) 1 and 24 hours after long-term sensitization training (*n*s = 10, 13 respectively except for 1-hour C/EBP where *n* = 11). Fold changes are calculated as the ratio of transcript from the trained side to the untrained side. Data are shown on a log scale, and the dotted line at 1 indicates no change (equal levels of transcript in the treated and control animal). * Indicates the mean-fold change is significantly different than 1 by a one-sample t-test (p<0.05).

We examined transcriptional changes 1 and 24 hours after long-term sensitization training (Figure 1A). We focused on 5 candidate plasticity genes which are rapidly up-regulated by *in vivo* 5-HT treatment, but which have not yet been tested for regulation during sensitization: CREB1, matrilin, antistasin, eIF3e, and BAT1 homolog.

We first confirmed the efficacy of our training protocol by examining both behavioral (24-hour group) and transcriptional measures (1 hour group). Behavioral measures taken in the 24-hour group confirmed prior reports [16]–[20] that the training protocol produces robust but unilateral long-term sensitization (Figure 1B). On the trained side, T-SWR duration increased substantially from pre-test (*M* = 13.0 s [9.1, 16.6], *SD* = 6.6) to post-test (*M* = 34.5 s [21.0, 48.0], *SD* = 23.4). In contrast, reflex durations were stable on the trained side from pre-test (*M* = 12.4 s [9.0, 14.9], *SD* = 5.1) to post-test (*M* = 12.4 s [8.8, 16.0], *SD*  = 6.2). The restriction of learning to the trained side was evident as a significant interaction term in a 2 (side: trained, untrained) x2 (time: pre-test, post-test) repeated-measures ANOVA (*F*(1,13)  = 17.1, *p* = 0.001, η^2^ = 0.57). Despite this substantial group effect, 1 of the 14 animals in this group did not exhibit long term sensitization (post-test duration was not longer than pre-test duration on the trained side). This non-responsive animal was not analyzed further, leaving 13 animals for the transcriptional analysis in the 24-hour group.

For the 1 hour group, animals were sacrificed before long-term sensitization could be confirmed behaviorally. We thus used the expression of C/EBP as a marker of training effectiveness. We confirmed [11] that the training protocol produces a rapid and robust increase in C/EBP (Figure 1C), with a mean fold-change (*M*) of 5.1 [2.4, 7.8] from the trained to the untrained side, a significant increase (*SD* = 3.9, *d* = 1.0, *t*(10) * = *3.42, *p* = 0.007). However, 1 of the 11 animals in this group was not transcriptionally responsive (expression of C/EBP was not higher on the trained side compared to the untrained side). Samples from this animal were not analyzed further, leaving 10 animals in the 1-hour group. Expression of H4, the housekeeping gene used to normalize expression of test transcripts, was similar on the trained and untrained sides for both time points (mean difference in cycle number  = 0.34, *t*<1).

With the efficacy of training confirmed, we examined the effects of training on the 5 candidate plasticity genes of interest (Figure 1C). Surprisingly, training did not produce strong and consistent effects on the expression of matrilin, antistasin, and BAT1 homolog. In the 1-hour group, no more than 6 of 10 animals exhibited altered expression in the expected direction (all *t*s<1.4; *M* = 1.4 [0.7, 2.2], *M* = 1.3 [0.7, 1.8], *M* = 1.5 [0.6, 2.4], respectively). Similarly, in the 24-hour group expression levels for these transcripts were similar between trained and untrained sides (all *t*s<1.2, *M* = 1.0 [0.5, 1.4], 1.0 [0.5, 1.4], 1.3 [0.7, 1.9], respectively).

For eIF3e, no regulation was evident 1 hour after training (*M* = 1.16 [0.6, 1.7], *SD* = 0.8, *t*<1, Figure 1C), with only 5 of 10 animals having increased expression on the trained side. In the 24-hour group, however, there was a consistent but weak trend towards up-regulation. Specifically, 10 of 13 animals showed higher expression of eIF3e on the trained side, but the mean fold-change was very modest (*M* = 1.1 [1.0, 1.3] and did not reach statistical significance (*SD* = 0.3, *d* = 0.4, *t*(11)  = 1.58, *p* = 0.14).

In contrast to the other transcripts of interest, CREB1 was strongly up-regulated 1 hour after training, with all 10 animals exhibiting higher expression on the trained side (*M*  = 2.0 [1.4, 2.6], *SD* = 0.83, *d* = 1.2, *t*(8)  = 3.70, *p* = 0.0049, Figure 1C). Surprisingly, however, the regulation of CREB1 was not persistent, as had been reported following 5-HT exposure to isolated ganglia [21]. Instead, only 6 of 13 animals in the 24-hour group had higher expression on the trained side, and overall expression levels were quite similar between sides (*M* = 1.2 [0.8, 1.6], *SD* = 0.7, *t*<1).

To ensure that the lack of persistent regulation in CREB1 was not due to poor sensitivity, we measured the expression of the *Aplysia* homolog of BiP/GRP78 (BiP) as a positive control. This transcript is known to exhibit delayed but persistent up-regulation after long-term sensitization training [22]. As expected, expression was not affected by training in the 1-hour group (*M* = 1.4 [0.8, 2.1], *SD* = 0.9, *t* = 1.42, *p* = 0.19, Figure 1C), but was strongly up-regulated in the 24-hour group (*M* = 3.1 [1.4, 4.7], *SD* = 2.7, *d* = 0.8, *t*(11)  = 2.42, *p* = 0.03).

To ensure regulation of C/EBP and CREB1 were specific to training, we measured levels of the *Aplysia* homolog of α-tubulin 2, a transcript not regulated by *in vivo* 5-HT treatment in *A. kurodai* [14]. In the 1-hour group, there was a modest but non-significant trend towards increased expression (*M* = 1.4 [0.9, 1.7], *SD* = 0.5, *t*(8)  = 2.08, *p* = 0.07, Figure 1C). In the 24-hour group, trained and untrained sides were similar in expression (*M* = 1.1 [0.8, 1.4], *SD* = 0.49, *t*<1). In addition, a set of animals given only sham shocks (touched with the shock wand but no current applied) showed no changes in T-SWR behavior just after training nor alterations in C/EBP levels (n = 3, data not shown).

### Transcriptional changes after in vivo serotonin exposure

**Figure 2:**
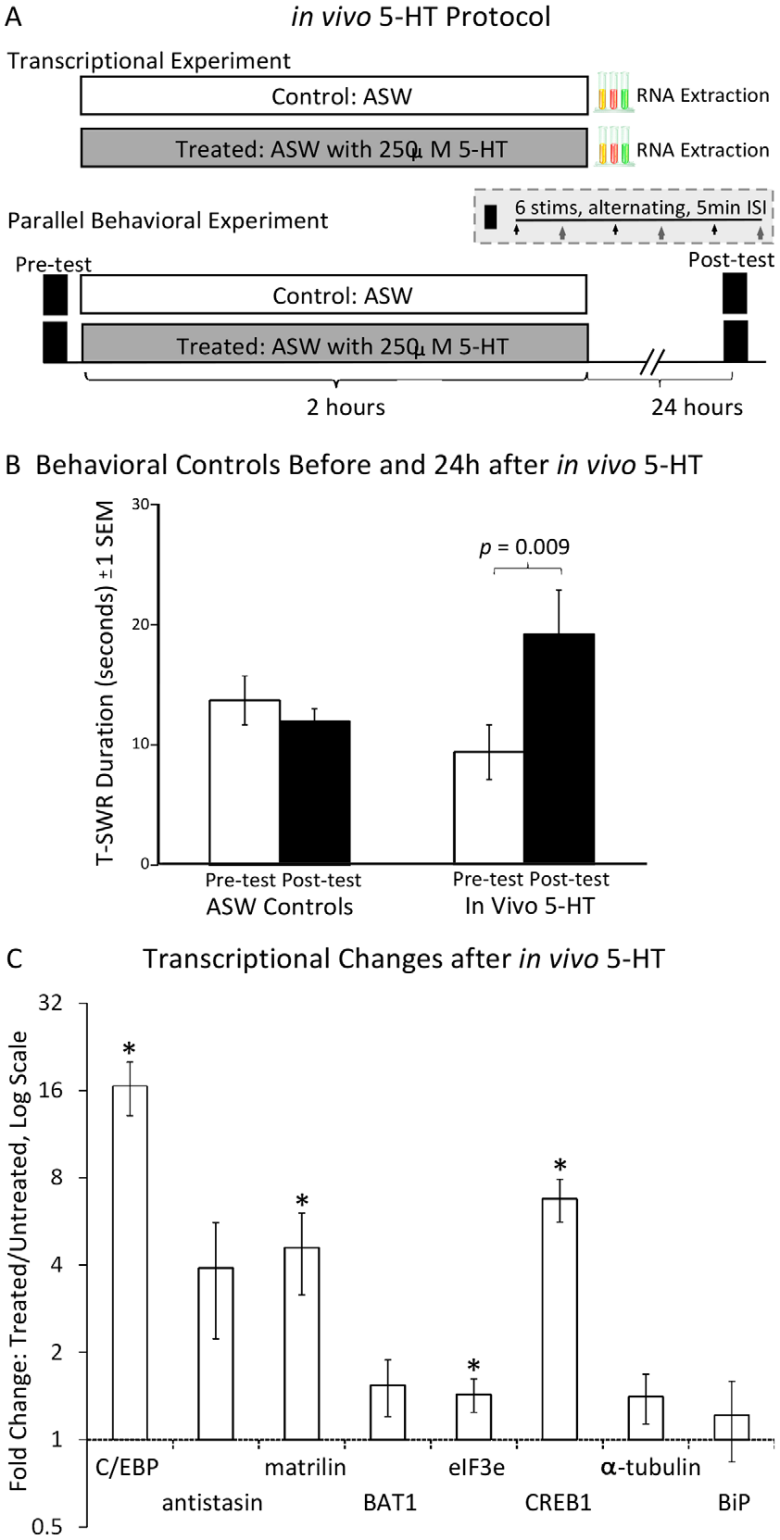
Transcriptional changes immediately following *in vivo* serotonin (5-HT) exposure. A. Protocol for *in vivo* 5-HT exposure. Experimental animals were immersed in artificial sea water (ASW) with 250 µM 5-HT for 2 hours; pleural ganglia were harvested for qPCR immediately afterwards. Each treated animal was matched with a control animal processed at the same time but immersed in ASW without 5-HT. To ensure this protocol produces long-term sensitization, a parallel behavioral experiment was conducted in which T-SWR durations were measured before (pre-test) and 24 hours after (post-test) treatment with either *in vivo* 5-HT or ASW. B. Mean T-SWR durations (±1 *SEM*) before and 24 hours after control ASW (n = 6) or *in vivo* 5-HT exposure (n = 8). T-SWRs were evoked via weak electrical shock to implanted electrodes in the tail and measured from the time of siphon contraction to the first sign of siphon relaxation. For each animal, pre-test and post-test responding was measured as the mean of 6 T-SWRs alternating between the left and right sides at a 5 minute ISI. The *p* value shown is for a paired t-test comparing pre-test and post-test responses within the treated group. The same comparison within the control ASW group was not significant. C. Mean transcriptional changes (± 1*SEM*) following *in vivo* 5-HT exposure (n = 10 pairs). Fold changes are calculated as the ratio of transcript in each treated animal versus its matched control. Data are shown on a log scale, and the dotted line at 1 indicates no change (equal levels of transcript in the treated and control animal). * Indicates the mean fold-change is significantly different than 1 by a one-sample t-test (p<0.05).

Results after long-term sensitization training indicate a surprising lack of regulation in several transcripts previously reported to be by altered by *in vivo* 5-HT. It may be, then, that *in vivo* 5-HT does not accurately recapitulate the transcriptional changes induced by behavioral training. To address this issue, we conducted an *in vivo* 5-HT experiment for direct comparison with the long-term sensitization experiment. We adopted the same treatment protocol (250 µM 5-HT for 2 hours, see Figure 2A) used to identify matrilin, eIF3e, BAT1 homolog, and antistasin as candidate plasticity genes in *A. kurodai* [14] and C/EBP in *A. californica* [9]. As with the previous experiment, however, we used *A. californica*.

We again confirmed training efficacy by measuring changes in C/EBP expression. As expected [9], this transcript was strongly up-regulated by *in vivo* 5-HT (*M* = 16.6 [8.7, 24.4], *SD* = 11.0, *d* = 1.4, *t*(8)  = 4.5, *p* = 0.002, Figure 2C). Every pair of animals tested was responsive (stronger C/EBP expression in treated vs. untreated animal), so no samples were screened. Again, expression of H4, which we used to normalize expression of other transcripts, was similar between conditions (mean difference in cycle number  = 0.042, *t*<1).

We also confirmed the behavioral efficacy of the training, this time with a set of parallel controls exposed to either *in vivo* 5-HT (n = 8) or sea water (n = 6). As expected (Figure 1B), T-SWR durations were stable in the control condition (at pretest: *M* = 13.7 s [8.1, 19.3], *SD* = 5.3; at post-test *M* = 12.0 s [9.1, 14.8], *SD* = 2.7) but rose sharply in every animal treated with 5-HT (at pretest: *M* = 9.4 s [5.1, 13.7], *SD* = 5.1; at post-test: *M* = 19.2 s [12.3, 26.1], *SD* = 8.2). An ANOVA confirmed a significant interaction between treatment and test (*F*(1,12)  = 10.5, *p* = 0.007, η^2^ = 0.47) due to the development of long-term sensitization in only the treated group. In a previous report [23], shorter duration (1.5 hours) *in vivo* 5-HT exposure at the same dose was not sufficient to produce statistically significant long-term sensitization. Presumably, the longer duration we used here accounts for this difference.

With treatment efficacy confirmed, we examined transcriptional regulation in the candidate plasticity genes of interest (Figure 2C). Consistent with the long-term sensitization experiment, CREB1 was strongly up-regulated by *in vivo* 5-HT. All 10 pairs tested showing stronger CREB1 expression in the treated animal (*M* = 6.8 [4.2, 9.3], *SD* = 3.6, *d* = 1.6, *t*(8)  = 5.1, *p* = 0.0007).

Also consistent with the long-term sensitization experiment, there did not seem to be consistent regulation of BAT1 homolog, antistasin, BiP, and α-tubulin 2 (Figure 2C). BAT1 homolog was down-regulated in only 3 of 10 pairs, and the mean fold-change indicated slightly *higher* expression on the trained side (*M* = 1.5 [0.7, 2.3], *SD* = 1.0, *t*(8)  = 1.6, *p* = 0.15). Expression of antistasin was quite variable, but this variability was independent of condition, with almost as many pairs showing a strong decrease in expression (3 pairs with fold-change ≤0.5) as showed a strong increase in expression (4 pairs with fold change ≥2). This result seems due to natural between-animal variability in the expression of antistasin, and statistical analysis did not indicate a reliable effect of 5-HT exposure (*M* = 3.9 [0.1, 7.7], *SD* = 5.3, *t*(8)  = 1.7, *p* = 0.12). Expression of BiP was similar in trained and untrained animals, as was expected for a transcript exhibiting delayed regulation after learning (*M* = 1.2 [0.8, 2.0], *SD* = 1.19, *t*<1). Finally, α-tubulin 2 expression was also unaffected (*M* = 1.4 [0.8, 2.0], *SD* = 0.9, *t*(8)  = 1.5, *p* = 0.17), as previously shown in *A. kurodai* [14].

In contrast to the long-term sensitization experiment, both matrilin and eIF3e were strongly and rapidly regulated by *in vivo* 5-HT (Figure 2C). For matrilin, expression was up-regulated in the trained animal in all 10 pairs tested (*M* = 4.6 [1.3, 7.9], *SD* = 4.6, *d* = 0.8, *t*(8)  = 2.5, *p* = 0.03). For eIF3e, there was a less consistent (7 of 10) pattern of regulation that just reached statistical significance (*M* = 1.4 [1.0, 1.9], *SD* = 0.60, *d* = 0.7, *t*(8)  = 2.3, *p* = 0.049).

To ensure that the lack of regulation observed for BAT1 homolog and antistasin were not due to insufficient experimental impact, we piloted an additional experiment with a stronger *in vivo* exposure of 500 μM 5-HT for 2 hours. In addition, this pilot work was conducted with wild-caught animals to ensure that findings generalize across animal sources. We found the same pattern of results with the pilot data for this higher dose. Expression of C/EBP was strongly up-regulated in every pair tested (*M* = 4.91 [3.3, 6.6], *SD* = 1.8, *d* = 2.1, *t*(4)  = 5.2, *p* = 0.003). In contrast, BAT1 homolog was not consistently regulated, with only 2 of 6 pairs showing the expected decrease, and a non-significant trend towards *up*-regulation (*M* = 1.16 [0.74, 1.6], *SD* = 0.45, *t*<1). Expression of antistasin again showed considerable variability unrelated to treatment (*M* = 1.89 [0.6, 3.1], *SD* = 1.3, *t*(4)  = 1.6, *p* = 0.17) with 3 pairs showing a decrease in expression and 3 pairs showing an increase in expression.

## Discussion

We used *A. californica* to measure the effects of both long-term sensitization training and *in vivo* 5-HT on expression of a set candidate plasticity genes: CREB1, matrilin, antistasin, eIF3e, and BAT1 homolog. These transcripts have been identified using *in vivo* 5-HT as potentially playing key roles in the initial transcriptional encoding of long-term sensitization memory. Surprisingly, we did not observe the predicted pattern of results for any of the transcripts tested. Antistasin and BAT1 homolog were not consistently regulated at any time point by any manipulation. Both matrilin and eIF3e were rapidly up-regulated by *in vivo* 5-HT, but not by long-term sensitization training. Only CREB1 showed the expected increase in expression 1 hour after training. However, CREB1 was not persistently regulated, as has been observed following treatments with 5-HT applied to isolated ganglia [21].

When interpreting negative results, experimental sensitivity is a key consideration. A frank lack of sensitivity can be ruled out, as we were able to detect up-regulation of two positive controls (C/EBP and BiP) as well as CREB1. In addition, power analysis indicates strong sensitivity for these experiments. We estimated effect sizes (*d*) from previous reports [14] on these transcripts to be at least 1.4, which agrees well with the range of effect sizes we observed in regulated transcripts (0.75 to 1.6). For this effect size, statistical power (sensitivity) is 0.97 for the smallest sample size we used (10), and recommended power levels (0.8) are maintained for effect sizes as small as 1.0. Thus, the negative results in these experiments do not necessarily indicate a true lack of regulation, but they do indicate that any regulation that does occur is likely to be of a relatively small magnitude compared to a) other known plasticity genes and b) previous estimates. A convenient way of integrating sensitivity into the interpretation of experimental results is through confidence intervals [24], which have been reported throughout. For example, we estimate that long-term sensitization produces a rapid regulation of BAT1 homolog that is between 0.7- to 1.7-fold of controls (95% CI), an estimate which includes the strong possibility of no regulation (fold change of 1) and which is almost certainly less than an estimated 2.7- to 8.3-fold change in C/EBP. Two caveats must be added to these considerations. First, only 2 times points were tested, so regulation could be occurring on a different time scale. Second, analyses were conducted on whole ganglia. Although sensitive at this level, regulation restricted to small subsets of neurons may not be detectable with this approach. Moreover, regulation of transcripts could be occurring in neurons outside the sampled ganglia.

Allowing for reasonable experimental sensitivity, this work provides an interesting comparison between long-term sensitization training and *in vivo* 5HT, which has proven a popular stand-in for actual behavioral training. These paradigms are known to be similar in causing a long-term increase in reflex duration [23] and a rapid increase in C/EBP protein [11], [12]. Our comparison over multiple transcriptional measures indicates, however, that *in vivo* 5-HT may not be an optimal model of long-term sensitization training. Of the 2 transcripts we tested that are up-regulated by sensitization training (C/EBP and CREB1), both are regulated by *in vivo* 5-HT (100% sensitivity). Of the 6 transcripts we tested which are not regulated by sensitization training, 2 (matrilin and eIF3e) were altered by *in vivo* 5-HT (67% selectivity). Although this suggests some caution in the use of *in vivo* 5-HT, this apparent lack of selectivity may only reflect a difference in experimental impact between treatments. Specifically, *in vivo* 5-HT involves sustained bilateral treatment of the entire organism whereas long-term sensitization involves transient unilateral stimulation over a restricted area of the body. Consistent with this interpretation, behavioral training produced smaller effect sizes in C/EBP and CREB1. In this case, it may be that behavioral training does regulate matrilin and eIF3e, but not to a detectable level. On the other hand, the within-subjects design enabled by the long-term sensitization protocol resulted in substantially lower variance in the expression of nearly every transcript, providing much greater experimental power than the *in vivo* 5-HT protocol. In addition, we found that *in vivo* 5-HT produces somewhat smaller behavioral effects than long-term sensitization training, which contrasts with its larger transcriptional impact. It seems possible, then, that the effects of *in vivo* 5-HT on matrilin and eIF3e represent effects of global 5-HT treatment that are not specific to long-term sensitization learning.

Across both paradigms we failed to observe consistent regulation of antistasin and BAT-1 homolog in *A. californica*. This contrasts with the results found in *A. kurodai*, in which these transcripts are rapidly and strongly increased by *in vivo* 5-HT treatment [14]. One possibility is that these transcripts are not actually regulated by experience. A more intriguing possibility is that this difference represents heterogeneity in the transcriptional mechanisms of memory between *A. californica* and *A. kurodai*. The possibility is supported by a recent large-scale comparison of EST sequences between these species [25]. This analysis showed that transcripts associated with signal transduction, and in particular with learning and memory, showed higher rates of evolutionary divergence between these species than those transcripts associated with cellular housekeeping. Indeed, diversity is apparent in the EST sequences across these species (see Supplemental Table 1), particularly for antistasin (90% sequence identity) and BAT1 homolog (89% sequence identify). These estimates of similarity are currently limited to the known EST sequences; the full-length mRNAs remain uncharacterized. In addition, there may be considerable variation in the regulatory regions for these genes, perhaps conferring different sensitivities to learning experiences. It seems possible, then, that differences in regulatory regions and/or transcription factor function could confer species-specific diversity in the transcriptional encoding of long-term memories. This would not be surprising, given that even subtle strain differences within a species are associated with profound differences in the molecular mechanisms of both plasticity and memory [26]–[29]. Direct comparison of transcriptional regulation across these species of *Aplysia* will be able to shed light on this issue.

Although we observed rapid up-regulation of CREB1 after both treatments, regulation after long-term sensitization training was transient, with no change in expression evident 24 hours after training. This result is surprising, as work using pulsed 5-HT exposure in isolated ganglia and cultured neurons has shown that CREB-1 is auto-regulating, promoting its own transcription both immediately and for at least 24 hours after activation [21] (though a more delayed onset of regulation has also been reported [30]). The long-term elevation of CREB1 transcription is important for consolidation of the long-term synaptic facilitation that accompanies repeated 5-HT exposure [21]. One possibility is that the long-term regulation of CREB1 is too subtle to detect following long-term sensitization training. Another is that this regulation may not occur in the pleural ganglia containing the sensory neuron cell bodies thought to mediate defensive withdrawal. This is an intriguing possibility, as it could suggest regulation in their post-synaptic targets in the pedal ganglia. We plan on directly exploring regional regulation of CREB1 at different time points following long-term sensitization training.

## Materials and Methods

### Animals

Animals (75–25 g) were obtained from the RSMAS National Resource for *Aplysia* (Miami, FL). For the pilot work reported with 500 μM *in vivo* 5-HT we used wild-caught animals (100–0 g) obtained from Marinus Scientific (Santa Barbara, CA).

Animals were maintained at 16°C, housed separately, fed dried seaweed twice a week, and kept on a 12 hr light-dark cycle. Animals were fed to satiation 2 days prior to experimental testing and then food deprived for the duration of the experiment. To eliminate the possibility of batch/shipment effects, animals from at least 2 different shipments were used for each experiment.

### Experimental Manipulations

A one-day long-term sensitization training protocol (Figure 1A) was conducted similarly to Wainwright et al.[20] but with a 90 mA stimulus rather than a 60 mA stimulus. We selected this stimulus amplitude after pilot-testing amplitudes of 30, 60, and 90 mA (n = 3 each) and finding that 90 mA produced stronger and more reliable elevations of both behavior and C/EBP expression. Training consisted of 4 rounds of noxious shock applied at 30 minute intervals to one side of the body with a hand-held electrode (WPI Constant-Current Stimulator, Sarasota, FL). Each round consisted of 10 pulses of 500 ms duration at a rate of 1hz and an amplitude of 90 ma AC. For transcriptional analysis, animals were harvested 1 or 24 hours after training. The 1 hour time point was selected because this is an interval during which expression of the immediate-early gene C/EBP is strongly up-regulated after long-term sensitization training [11]. For the 24-hour time point, behavioral measures were collected (see below) by an observer blind to the experimental condition. One of the advantages of this training protocol is that both the induction and expression of sensitization is restricted to one side of the body [16]–[20], enabling each animal to serve as its own control for both behavioral and transcriptional experiments (trained vs. untrained sides).

*In vivo* 5-HT exposure (Figure 2A) was conducted just as previously conducted to identify candidate plasticity genes in *A. kurodai* [14] and *A. californica* [9]. Specifically, treated animals were placed in 250 ml of 250 μM 5-HT (H7752, Sigma, Saint Louis, MO) dissolved in artificial sea water (ASW). Each treated animal was matched with a control animal from the same shipment. Control animals were placed in the same volume of ASW for the same amount of time, but without 5-HT. Exposures lasted 2 hours, followed by immediate dissection for qPCR analysis. A set of parallel behavioral controls was tested 24 hours after exposure to ensure the protocol produced long-term sensitization.

### Behavioral Measurement

As a behavioral outcome, we measured the duration of the tail-elicited siphon-withdrawal reflex (T-SWR). This reflex is triggered by stimulation of the tail and results in a defensive withdrawal of the siphon, an exhalent respiratory structure. The procedure for evoking and measuring T-SWR behavior was adapted from Scholz and Byrne [19]. Briefly, animals were implanted with pairs of Teflon-coated silver wire electrodes (0.005 inch diameter, A-M Systems, Sequim, WA). T-SWRs were evoked via mild electrical shocks applied to the left or right pair of electrodes (60Hz AC, 50 ms; amplitudes were set at 2x threshold and ranged from 2-10 mA). T-SWR behavior was measured as the duration of withdrawal from the moment of stimulation to the first sign of siphon relaxation. To characterize changes in T-SWR duration, pre-test and post-test responsiveness was characterized by a series of 6 responses evoked on alternating sides of the body at a 5-min ISI. For the long-term sensitization experiment, scores were split by side (trained vs. untrained) each measured as the average of 3 T-SWR responses. For the *in vivo* 5-HT experiment, which impacts the entire animal, all 6 responses were averaged together.

### Isolation and Processing of Pleural Ganglia RNA

To analyze transcription, pleural ganglia RNA was isolated. These ganglia were selected because they contain the cell bodies of the tail-sensory neurons which are thought to serve as an important site for the neural plasticity underlying behavioral sensitization [31]. Briefly, animals were anesthetized with an injection of isotonic MgCl_2_ (50% of body weight), and an incision was then made along the ventral midline to expose the CNS. For the *in vivo* 5-HT experiment, the left and right pleural ganglia were harvested together. For the behavioral sensitization experiment, the left and right pleural ganglia were harvested separately. As dissection can alter gene expression [9] (Alberini, C. M. 1994 ref_num764)ref_af, we extracted ganglia rapidly (<5 minutes per animal) and transferred them immediately to RNAlater on ice (Ambion, Austin, TX).

Tissue was homogenized and RNA extracted using Trizol (Invitrogen, Carlsbad CA) and RNeasy Mini Kit (Qiagen, Valencia CA). Quantity and quality of RNA was assessed using the NanoDrop 2000 (Thermo Scientific, Wilmington DE). RNA was reverse transcribed using oligo dT primes with First Strand cDNA synthesis kit (Fermentas, Glen Burnie MD).

### Quantitative PCR

Expression levels were analyzed by qPCR using Sybr Green and the MyIQ real time PCR system (Bio-Rad, Los Angeles CA). For transcripts first identified in *A. kurodai*, we used the EST or mRNA reported for that species to identify the best-matching EST or mRNA sequence in *A. californica* and designed primers based on the matched *A. californica* sequence (see Table 1). All primers were validated for correct PCR efficiency and were then confirmed for selective amplification through sequencing of their PCR products. qPCR samples were analyzed in triplicate and the relative amounts of each transcript were determined using the standard curve method. Expression levels were normalized to levels of histone H4, a transcript not regulated by *in vivo* 5-HT treatment[32]. To quantify regulation, a fold-change score was calculated for each animal/pair as the ratio of expression from treated to untreated. Statistical analyses were conducted on fold-change scores using a one-sample *t*-test against the expected value of 1 for the null hypothesis. Mean fold-change scores are reported with 95% confidence intervals in brackets.

## Supporting Information

Table S1 Transcript accession numbers and primer sequences with homologs in *A. kurodai* where available.
(PDF)
