# Supplementary material for: Transcriptional Changes following Long-Term Sensitization Training and In Vivo Serotonin Exposure in Aplysia californica
Source: PLoS One. 2012 Oct 9;7(10):e47378. doi: 10.1371/journal.pone.0047378 (PMC3467254; doi:10.1371/journal.pone.0047378)
Supplement: Table S1 — Transcript accession numbers and primer sequences with homologs in A. kurodai where available. (PDF) [file pone.0047378.s001.pdf]

Table S1: Transcript accession numbers and primer sequences with homologs in *A. kurodai* where available

Transcript	***A. kurodai*** **EST or mRNA**	***A. californica*** **EST or mRNA**	**Similarity[1]**	***A. Californica*** **Forward Primer** / ***A. Californica*** **Reverse Primer**
antistasin	EY417467	GD231438	89.80%	AAATGTTCCGGACGAGAATG
	(658 bp)	(727 bp)	(582/648)	CACACGTGTTGCATCCATTT
matrilin	EY418286	EB307354	91.70%	TCACGCTCACGTCTTCAAAC
	(618 bp)	(436 bp)	(400/436)	ATCGGCATAGCTGATGGAAC
BAT1 Homolog	EY418548	GD232017	88.80%	TGCCATGGCTGAAGACAGT
	(337 bp)	(780 bp)	(284/320)	CTGAACTGTGGATGGACACG
eIF3e	EU791459	GD215078	97.20%	AGAACCTGGGTGATCCACTG
	(1317 bp)	(775 bp)	(745/767)	GACACGTCGTCTGGATAGCA
CREB1	N/A[2]	Bartsch et al., 1998[6]	N/A	AGTCAAAGTCAAGACGGCCACGA
				TCCACCCGTGGAGTTTGAGGCT
C/EBP	N/A[2]	U00994.1	N/A	GCAACTCAGCAACGCAACAAATGC
				TTTAGCGGAGATGTGGCATGGAGT
α-Tubulin 2	EY417749	AF481056	95.60%	TCGTTGATCTCGTGCTTGAC
	(395 bp)	(1945 bp)	(350/366)	GCTTGGACTTCTTGCCGTAG
BiP/GRP78	N/A[2]	NM_001204652.1		ACGGCTGCTGACAACCAGCC
				ACACCACGAGGAGCTGGGGG

1: Similarity scores based on mRNA seuquence alignment using LFASTA (http://pbil.univ-lyon1.fr/lfasta.php)

2: N/A indicates that no mRNA or EST for that transcript has been described yet to *A. kurodai*

3: Sequence available in this reference but does not seem to have been catalogued in GenBank yet
